# Exploration of the influence of *GOLGA8B* on prostate cancer progression and the resistance of castration-resistant prostate cancer to cabazitaxel

**DOI:** 10.1007/s12672-024-00973-7

**Published:** 2024-05-10

**Authors:** Haopeng Li, Xin’an Wang, Menghe Zhai, Chengdang Xu, Xi Chen

**Affiliations:** 1 Department of Urology, Tongji Hospital, School of Medicine, Tongji University, 389 Xincun Road, Shanghai, 200065 China; 2 Department of Urology, Jiaxing Second Hospital, 397 North Huancheng Road, Jiaxing, 314000 Zhejiang China

**Keywords:** Castration-resistant prostate cancer, *GOLGA8B*, Cabazitaxel, Cancer development

## Abstract

Castration-resistant prostate cancer (CRPC) represents the final stage of prostate cancer (PCa). Cabazitaxel, a taxane chemotherapy drug, is used in treating CRPC. However, patients with CRPC eventually develop resistance to cabazitaxel, and the underlying mechanism remains unclear. Here, we aimed to investigate potential genetic alterations that may play a role in CRPC resistance to cabazitaxel. Using microarray data from the GSE158494 dataset, we identified ten critical genes (*CXCL8*, *ITGB8*, *CLIP4*, *MAP1B*, *WIPI1*, *MMP13*, *CXCL1*, *C1S*, *GOLGA8B*, and *CXCL6*) associated with CRPC cell resistance to cabazitaxel. The potential function of these key genes in PCa progression was analyzed using different databases, including Gene Expression Omnibus (GEO), The Cancer Genome Atlas (TCGA), and Chinese Prostate Cancer Genome and Epigenome Atlas (CPGEA). Our findings revealed altered expression of these genes in the development of PCa. Furthermore, *CXCL1* and *GOLGA8B* were found to influence the disease-free survival (DFS) status of patients with PCa, with *GOLGA8B* affecting the overall prognosis in patients with PCa. Additionally, *GOLGA8B* expression was associated with the infiltration of various immune cells in PCa, and it was upregulated in clinical PCa and CRPC samples. Through CCK-8 assays, we established that *GOLGA8B* could influence the sensitivity of CRPC cells to cabazitaxel and docetaxel. In conclusion, we identified *GOLGA8B* as a crucial gene that influences PCa progression and contributes to CRPC resistance to cabazitaxel.

**Supplementary Information:**

The online version contains supplementary material available at 10.1007/s12672-024-00973-7.

## Introduction

Prostate cancer (PCa) is a prevalent malignancy, primarily affecting older men, with an incidence rate exceeding 20%. It ranks as the second most frequent cause of cancer-related death in men in the United States of America [1]. China is experiencing a rapid increase in PCa incidence and mortality rates [2]. Currently, a range of effective treatments is available for PCa, with androgen deprivation therapy (ADT) being the preferred first-line treatment [3]. However, after undergoing ADT, these patients ultimately develop castration-resistant prostate cancer (CRPC) [4]. CRPC can be managed through various therapeutic modalities, including chemotherapy [5]. Taxane-based chemotherapeutic agents such as docetaxel have been used in CRPC treatment [6]. However, patients with CRPC treated with docetaxel eventually develop resistance to the drug, necessitating the use of alternative taxane drugs to address docetaxel-resistant CRPC.

Cabazitaxel belongs to the taxane family of drugs. It can bind tubulin and promote microtubule assembly by stabilizing them and preventing their depolymerization, which consequently interferes with cell division and results in cell death [7, 8]. Cabazitaxel has been shown to exert cytotoxic activity and retard the growth of several docetaxel-resistant tumor cells [9]. Furthermore, the roles of cabazitaxel in inhibiting human xenograft tumor growth [10] and in treating CRPC [11, 12] have been reported. Despite the efficacy of cabazitaxel in treating CRPC, patients ultimately become resistant to cabazitaxel, but the mechanism underlying this resistance remains unclear.

Genetic alterations have emerged as significant contributors to resistance to chemotherapeutic drugs in PCa. Studies have shown the pivotal role of genetic alterations as a fundamental factor underlying chemotherapy resistance in PCa, particularly for drugs like docetaxel and vinblastine [13–15]. Furthermore, research has highlighted that certain gene mutations can cause resistance to cabazitaxel in PCa [16, 17]. Consequently, investigating alterations in gene expression becomes crucial for elucidating the mechanisms underlying resistance to cabazitaxel in CRPC. In the present study, the GSE158494 dataset retrieved from the Gene Expression Omnibus (GEO) database was utilized to determine the upregulated genes in cabazitaxel-resistant CRPC cells. We identified ten upregulated hub genes that may be important for CRPC cells resistant to cabazitaxel. Furthermore, we singled out one of the ten hub genes, *GOLGA8B*, which can affect PCa occurrence, progression, and prognosis. We hypothesized that *GOLGA8B* can influence PCa development and cabazitaxel resistance simultaneously. Thus, we selected *GOLGA8B* for further study. *GOLGA8B* was identified as being upregulated in clinical PCa and CRPC specimens, demonstrating its effect on the sensitivity of CRPC cells to both cabazitaxel and docetaxel. This investigation reinforces the pivotal role of *GOLGA8B* in the development of PCa and resistance of CRPC to cabazitaxel.

## Materials and methods

### Data collection

This study involved the analysis of multiple datasets retrieved from various sources. We utilized the GSE158494 dataset, containing microarray data derived from cabazitaxel-sensitive and -resistant DU145 and PC-3 CRPC cells. Genes showing differential expression between cabazitaxel-sensitive and -resistant PC-3 and DU145 cells were considered hub genes crucial for the occurrence of cabazitaxel resistance. Additionally, two more datasets, GSE33455 and GSE36135, were acquired, both providing microarray data of docetaxel-resistant CRPC cells. These datasets were sourced from the Gene Expression Omnibus (GEO) database (http://www.ncbi.nlm.nih.gov/geo/). Furthermore, we analyzed the GSE35988 dataset, encompassing information on patients diagnosed with PCa and CRPC. In addition to these datasets, patient data from two distinct databases were examined: The Cancer Genome Atlas (TCGA; http://cancergenome.nih.gov/) and Chinese Prostate Cancer Genome and Epigenome Atlas (CPGEA; http://www.cpgea.com).

### Data handling

Raw microarray data retrieved from various databases underwent normalization using the “limma” package in the R software. During this process, the probe ID was replaced with the corresponding gene ID. Genes meeting the criteria of |log_2_FC > 1| and *P* < 0.05 were classified as cabazitaxel resistance-related genes. Additionally, clinical data were obtained from diverse databases.

### Pathway analysis

Pathway analysis was conducted through Gene Ontology (GO) and Kyoto Encyclopedia of Genes and Genomes (KEGG) analyses. The online tool Metascape, accessible at http://metascape.org/, was used for this analysis, and a bubble map was generated using the “ggplot2” R package.

### Survival analysis

The online web tool “GEPIA” facilitated the exploration of the correlation between cabazitaxel resistance-related genes and the disease-free survival (DFS) status in PCa patients. Univariate and multivariate Cox regression analyses were performed to determine the variables necessary to develop the nomogram. According to the median gene expression of each gene in PCa patients from TCGA database, patients were divided into high and low gene expression groups. Additionally, the “forest plot” package in the R software was employed to display *P*-values, hazard ratios (HRs), and 95% confidence intervals (CIs) for each variable. Subsequently, a nomogram was constructed based on the outcomes of the multivariate Cox proportional hazards analysis, serving as a predictive tool for overall recurrence.

### Immune infiltration analysis

Gene Set Cancer Analysis (GSCA; http://bioinfo.life.hust.edu.cn/GSCA/#/) is a public online web tool used to download the immune cell infiltration information of PCa patients from TCGA database. We first downloaded this immune infiltration information from GSCA. Using data from TCGA database, Spearman correlation analysis was conducted to evaluate the relationship between the expression of cabazitaxel resistance-related genes and immune cell infiltration (Additional file 1).

### Clinical specimen collection

Specimens were obtained from individuals diagnosed with PCa and CRPC at Tongji Hospital, School of Medicine, Tongji University. The collection procedure was approved by the Ethics Committee of Tongji Hospital, School of Medicine, Tongji University (SBKT-2021-220). Comprehensive information about the study was provided to all participating patients, and they provided informed consent for the utilization of their samples in the research.

### Cell culture and drug treatment

PCa cell lines were obtained from the Chinese Academy of Science Cell Bank (Shanghai, China). The human CRPC cell lines PC-3 and DU145 were cultured in Roswell Park Memorial Institute (RPMI) 1640 media (Sigma, Darmstadt, Germany; Catalog No. R8758) containing 10% fetal bovine serum (FBS; Gibco, Thermo Fisher Scientific, Waltham, MA, USA, Catalog No. 10091). Cell cultures were maintained at 37 ℃ in a humidified atmosphere with 5% CO_2_ and 95% air. Docetaxel and cabazitaxel were acquired for the study (SelleckChem, Houston, TX, USA, Catalog No. S1148 and S3022, respectively). The CRPC cells were exposed to docetaxel or cabazitaxel at a dose of 2 nmol/L for 24 h. Furthermore, both PC-3 and DU145 cells were treated with docetaxel (2 nmol/L) for 2 weeks to induce resistance. Upon observing that docetaxel did not affect cell growth, we concluded that these cells were resistant to docetaxel (Additional file 2).

### Cell transfection and lentivirus production

Cell transfection experiments were conducted using Lipofectamine 2000 (Thermo Fisher Scientific, Catalog No. 11668019). Specifically, shGOLGA8B lentivirus was designed to facilitate specific gene knockdown. Gene-specific shRNAs and a control lentivirus (shControl) were procured from Youze Biotechnology Company (Hunan, China).

### RNA extraction and qRT-PCR

Total RNA was isolated from clinical samples and CRPC cell lines using the TRIzol reagent (Sigma-Aldrich, St. Louis, MO, USA, Catalog No. T9424). Subsequently, cDNA synthesis was performed using a reverse transcription kit (Advantage^®^ RT-for-PCR Kit, Takara Bio Inc., Kusatsu, Japan, Catalog No. 639505). Gene expression analysis was conducted using a real-time PCR kit (TB Green^®^ Premix Ex Taq^™^ II, Takara Bio Inc., Catalog No. RR420A) following the manufacturer’s instructions. The PCR primer sequences for *GOLGA8B* and *GAPDH* (reference gene) are listed in Table 1. mRNA expression was quantified using the 2^−ΔΔCt^ technique.

**Table 1:** The primers used in the qRT-PCR

Gene name	Primer sequence
GOLGA8B	Forward: 5′-TGCCTCCCAGGTTCAAGCAA-3′
	Reverse: 5′-CACGTCAGCCAAATCCCAGC-3′
GAPDH	Forward: 5′-GGAGCGAGATCCCTCCAAAAT-3′
	Reverse: 5′-GGCTGTTGTCATACTTCTCATGG-3′

### Antibodies

Rabbit polyclonal anti-GOLGA8B antibody (Catalog No. ab155806) and rabbit monoclonal anti-GAPDH antibody (Catalog No. ab9485) were purchased from Abcam UK (Cambridge, UK).

### Western blotting

Proteins were extracted from tissues and cell lines using RIPA lysis buffer. Following extraction, protein samples were processed with treated with Dual Color Protein Loading Buffer (Thermo Fisher Scientific). Protein separation was achieved through SDS-PAGE using a 10% gel, followed by protein transfer onto nitrocellulose membranes sourced from Merck KGaA (Darmstadt, Germany). The Protein-Free Rapid Blocking Buffer (Thermo Fisher Scientific) was used to block the membranes. The membranes were exposed to primary antibodies against GOLGA8B (dilution 1:1000) and GAPDH (dilution 1:1000; Abcam UK) overnight at 4 ℃. The following day, the membranes were washed thrice using 1 × TBST (10 min/cycle) and then incubated at room temperature for 1.5 h with the corresponding secondary antibody [Catalog No. A0208, HRP-labeled Goat Anti-Rabbit IgG (H + L), obtained from Beyotime Biotechnology, Shanghai, China]. Finally, after exposing the membranes to X-ray film, protein identification was conducted.

### Cell proliferation assay

The Cell Counting Kit 8 (CCK 8; Dojindo, Japan) was used to assess the cell proliferation capacity. Briefly, cells were placed in 96-well plates at a density of 3000 cells/well. They were then cultivated for specific lengths of time (0, 24, 48, or 72 h) in 200 µL of RPMI 1640 supplemented with 10% FBS. Subsequently, cell viability was determined using the CCK8 assay following the provided protocol. A spectrophotometer (LD942, Beijing, China) was used to measure the absorbance at 450 nm.

### Statistical analysis

R v4.0.3 (Institute for Statistics and Mathematics, Vienna, Austria; https://www.r-project.org) was used to analyze the matrix data. The Wilcoxon test was applied for comparisons between two groups, and the Kruskal–Wallis test was utilized for comparisons involving more than two groups. Statistical metrics included *P* values, 95% CIs, and HRs. Statistical significance was set at *P* < 0.05 (two-tailed).

## Results

### Identification of ten upregulated hub genes in cabazitaxel-resistant CRPC cells

**Fig. 1:**
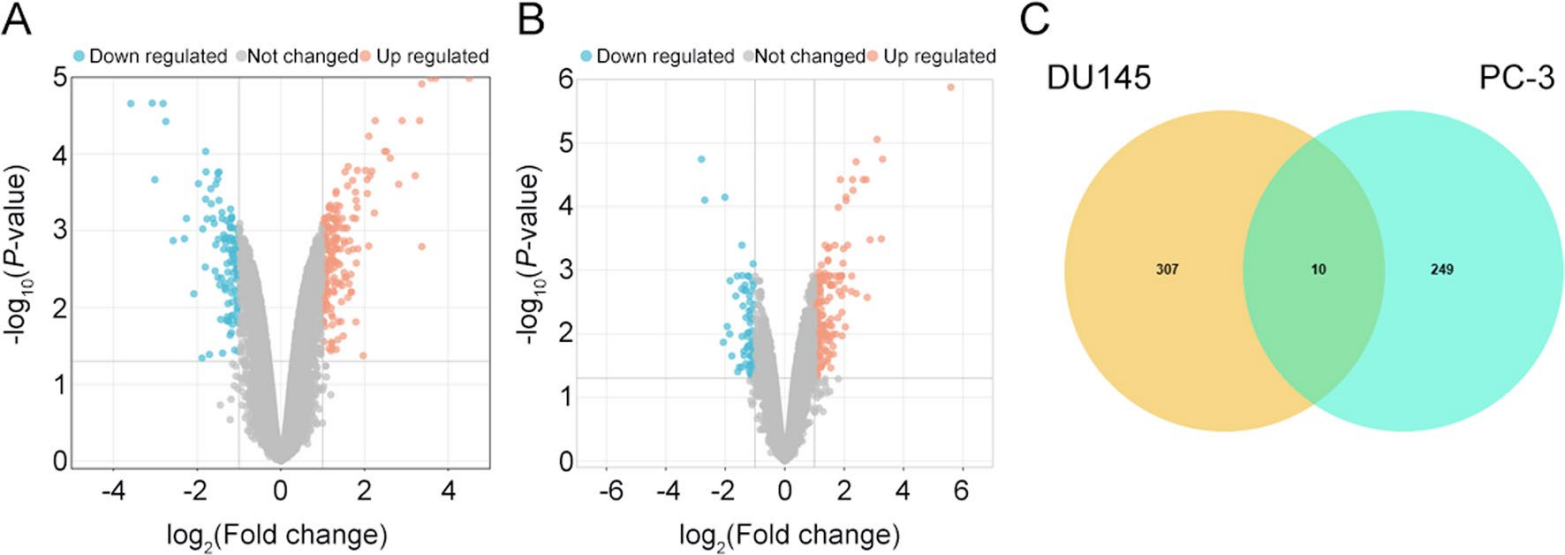
Ten cabazitaxel resistance-related genes were identified from the GSE158494 dataset. **A** Volcano map reflects the differently expressed genes between cabazitaxel-sensitive and -resistant DU145 cells from the GSE158494 dataset. **B** Volcano map reflects the differently expressed genes between cabazitaxel-sensitive and -resistant PC-3 cells from the GSE158494 dataset. **C** Ten genes associated with cabazitaxel resistance from both DU145 and PC-3 cells

To uncover the critical genes involved in the development of CRPC resistance to cabazitaxel, we conducted an analysis using the GSE158494 dataset. This dataset comprised microarray data from both cabazitaxel-sensitive and -resistant DU145 and PC-3 CRPC cells. The generation of volcano plots facilitated the visualization of differential gene expression between cabazitaxel-sensitive and -resistant CRPC cells (Fig. 1A, B). This analysis revealed the upregulation of ten hub genes in cabazitaxel-resistant cells compared with that in the sensitive cells, with no detection of downregulated genes (Fig. 1C).

### Pathways enriched in association with cabazitaxel resistance-related hub genes

**Fig. 2:**
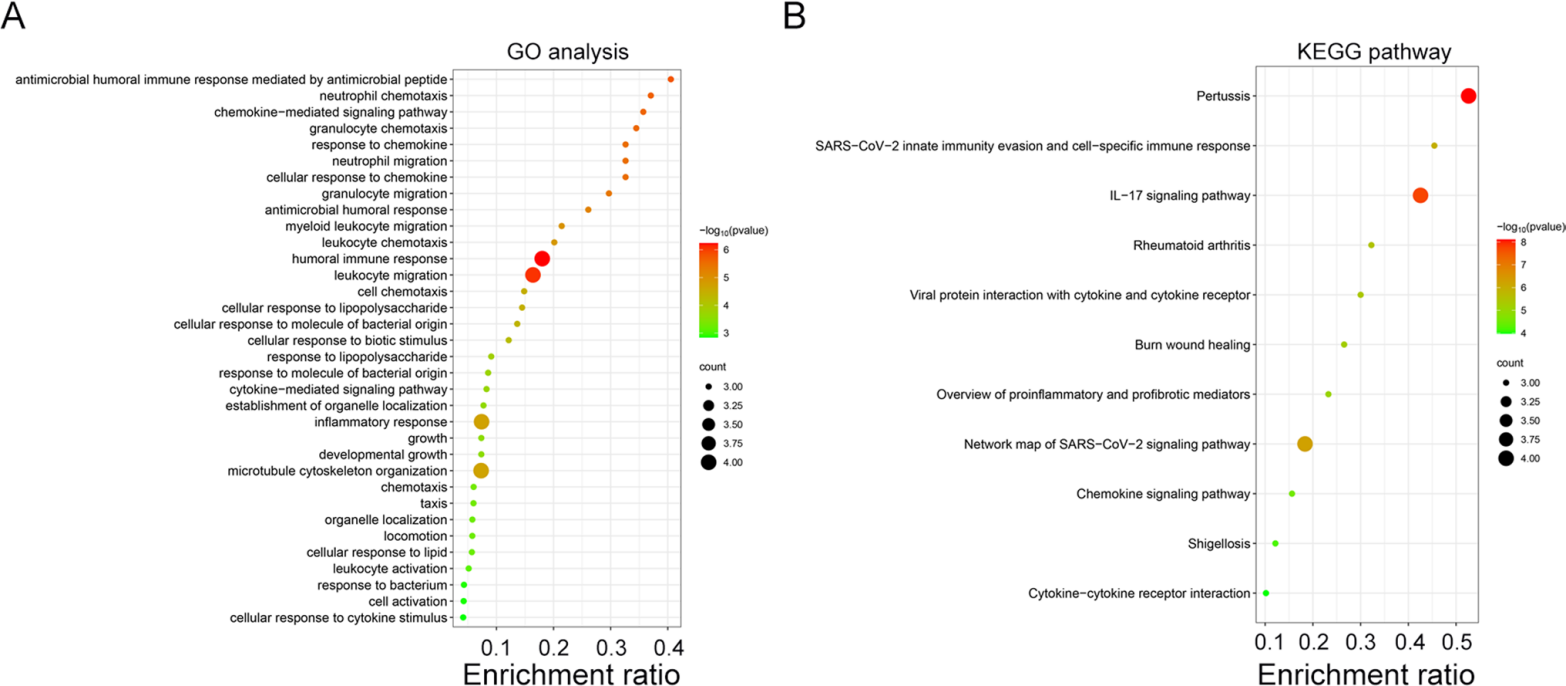
Pathways of cabazitaxel resistance-related genes enriched in (**A**) GO analysis and **B** KEGG pathways

We focused on identifying pathways associated with cabazitaxel resistance-related genes, using GO and KEGG pathway analyses. Bubble maps were constructed to illustrate the pathways exhibiting enrichment. We defined a pathway with the greatest number of enriched genes and the minimum *P*-value as the most important one. In GO analysis, notable enrichment was observed in the humoral immune response pathway (Fig. 2A). Furthermore, KEGG pathway analysis revealed a predominant enrichment in the IL-17 signaling pathway (Fig. 2B). These findings strongly imply that the hub genes may play a role in cabazitaxel resistance by regulating the immune response.

### Expression analysis of cabazitaxel resistance-related genes in PCa patients

**Fig. 3:**
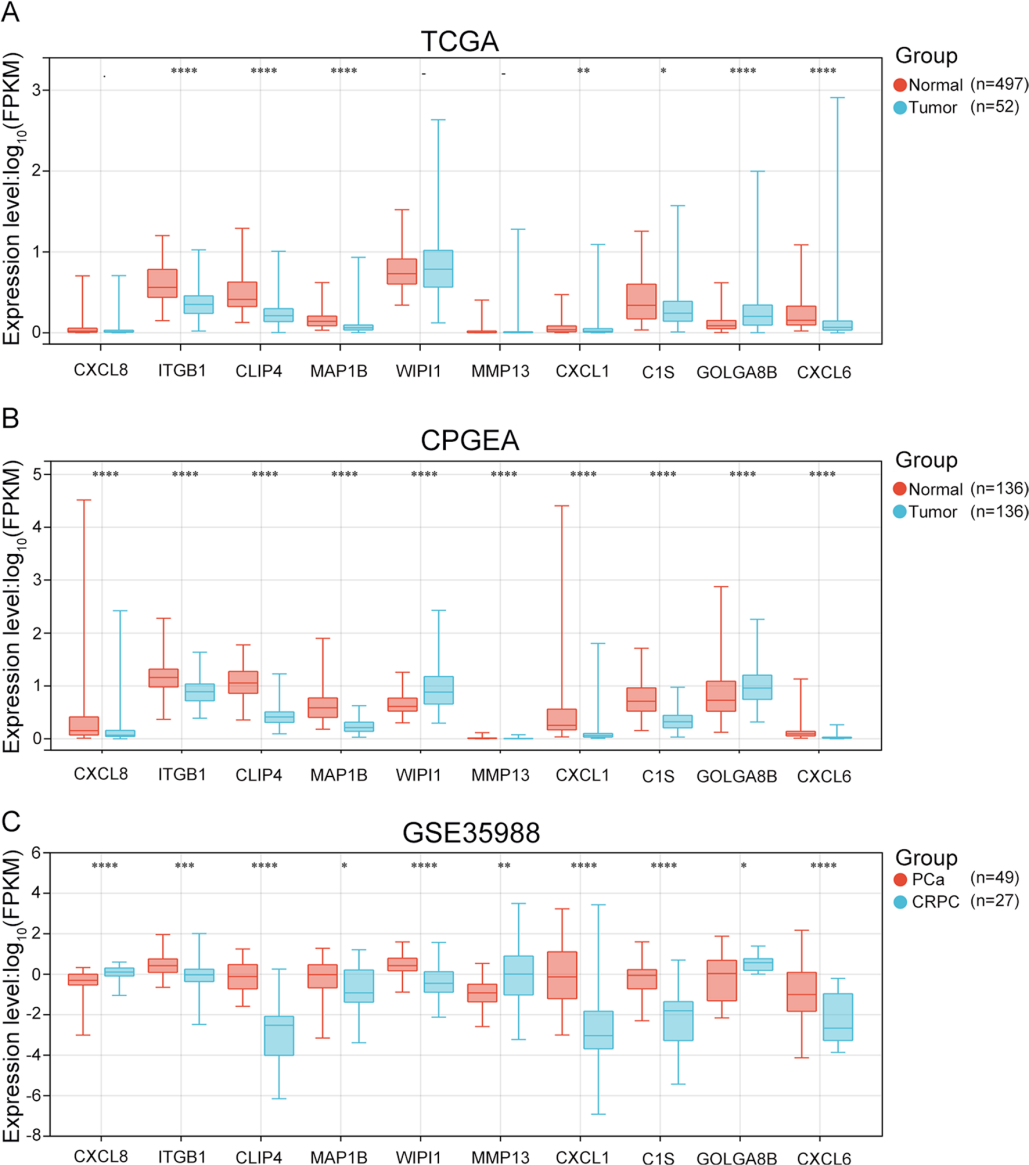
Expression of cabazitaxel resistance-related genes in PCa and CRPC patients from different databases. Expression of ten cabazitaxel resistance-related genes in PCa patients from **A** TCGA and **B** CPGEA databases. **C** Expression of cabazitaxel resistance-related genes in PCa and CRPC patients from the GSE35988 dataset. “–” represents no statistical differences. *P < 0.05, **P < 0.01, ***P < 0.001, ****P < 0.0001

To examine whether the identified cabazitaxel resistance-related genes from CRPC cells alter their expression upon the onset of PCa, we assessed the data acquired from TCGA, comparing the expression profiles of PCa patients and healthy individuals. Among the ten identified hub genes, *GOLGA8B* was found to be upregulated (*P* < 0.0001) in PCa tissues, whereas *ITGB1*, *CLIP4*, *MAP1B*, *CXCL1*, *C1S*, and *CXCL6* exhibited downregulation upon PCa onset (*P* < 0.05; Fig. 3A). To account for population diversity, since TCGA database primarily includes data from the Western population, the analysis was extended to the CPGEA database, which includes data from Chinese PCa patients. In this dataset, *WIPI1* and *GOLGA8B* (*P* < 0.0001) were upregulated in Chinese PCa patients, whereas the other genes were downregulated (*P* < 0.0001; Fig. 3B). Furthermore, any alteration in the expression of these genes during CRPC development was assessed by analyzing the GSE35988 dataset from the GEO database, which contains data from both PCa and CRPC patients. This analysis indicated that *CXCL8*, *MMP13*, and *GOLGA8B* were upregulated in CRPC samples (*P* < 0.05), whereas the other genes exhibited downregulation (*P* < 0.01; Fig. 3C). These findings collectively suggest that these cabazitaxel resistance-related genes may play pivotal roles in the onset and progression of both PCa and CRPC.

### Impact of CXCL1 and GOLGA8B on the survival status of PCa patient

**Fig. 4:**
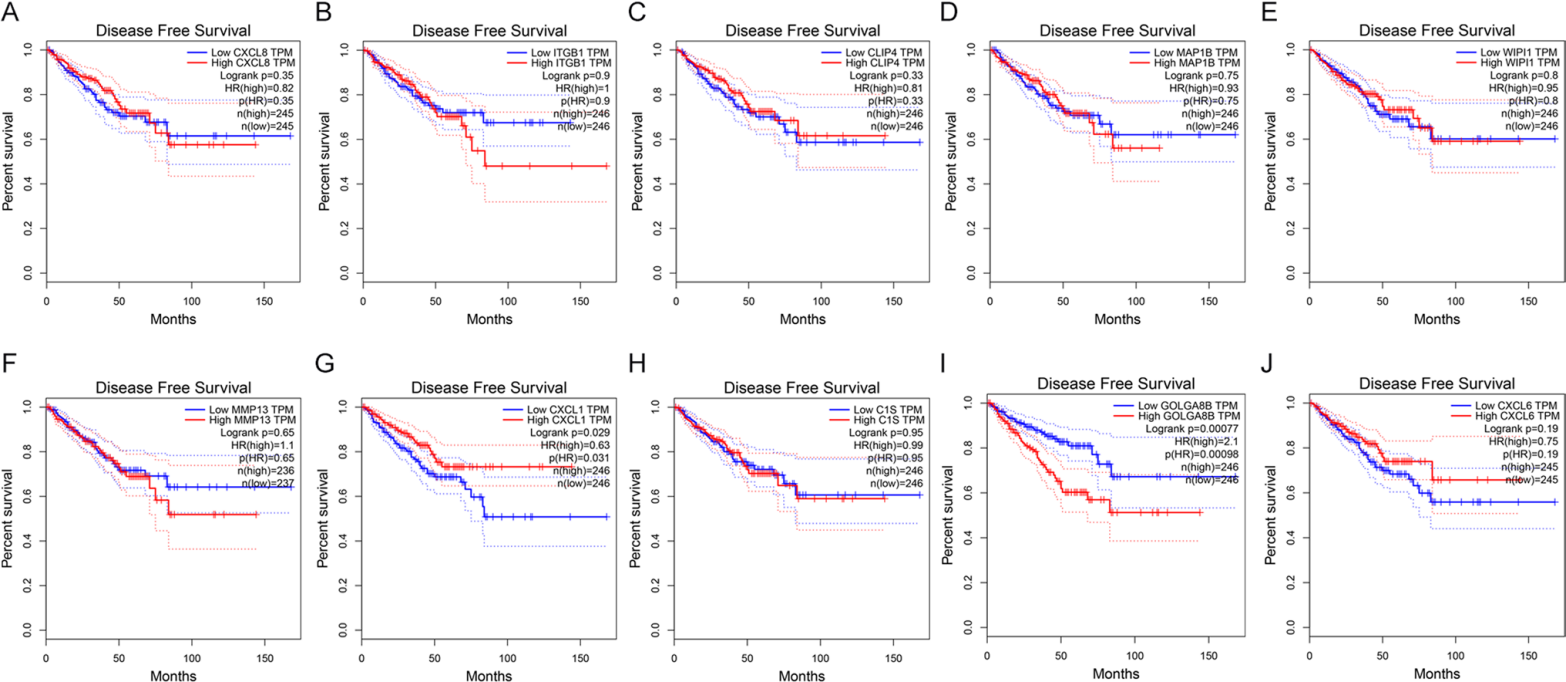
Expression of cabazitaxel resistance-related genes in relation to the DFS status of PCa patients from TCGA database. (**A**) *CXCL8* (**B**) *ITGB1* (**C**) *CLIP4* (**D**) *MAP1B* (**E**) *WIPI1* (**F**) *MMP13* (**G**) *CXCL1* (**H**) *C1S* (**I**) *GOLGA8B* (**J**) *CXCL6*

We investigated the potential impact of these hub genes on the survival outcomes of PCa patients. Using the online tool GEPIA, the correlation between the expression levels of the ten hub genes and the DFS status of PCa patients was assessed. The expression levels of *CXCL1* (*P* = 0.031) and *GOLGA8B* (*P* = 0.00077) exhibited correlations with the DFS status of PCa patients (Fig. 4).

### Impact of GOLGA8B on the prognosis of PCa patients

**Fig. 5:**
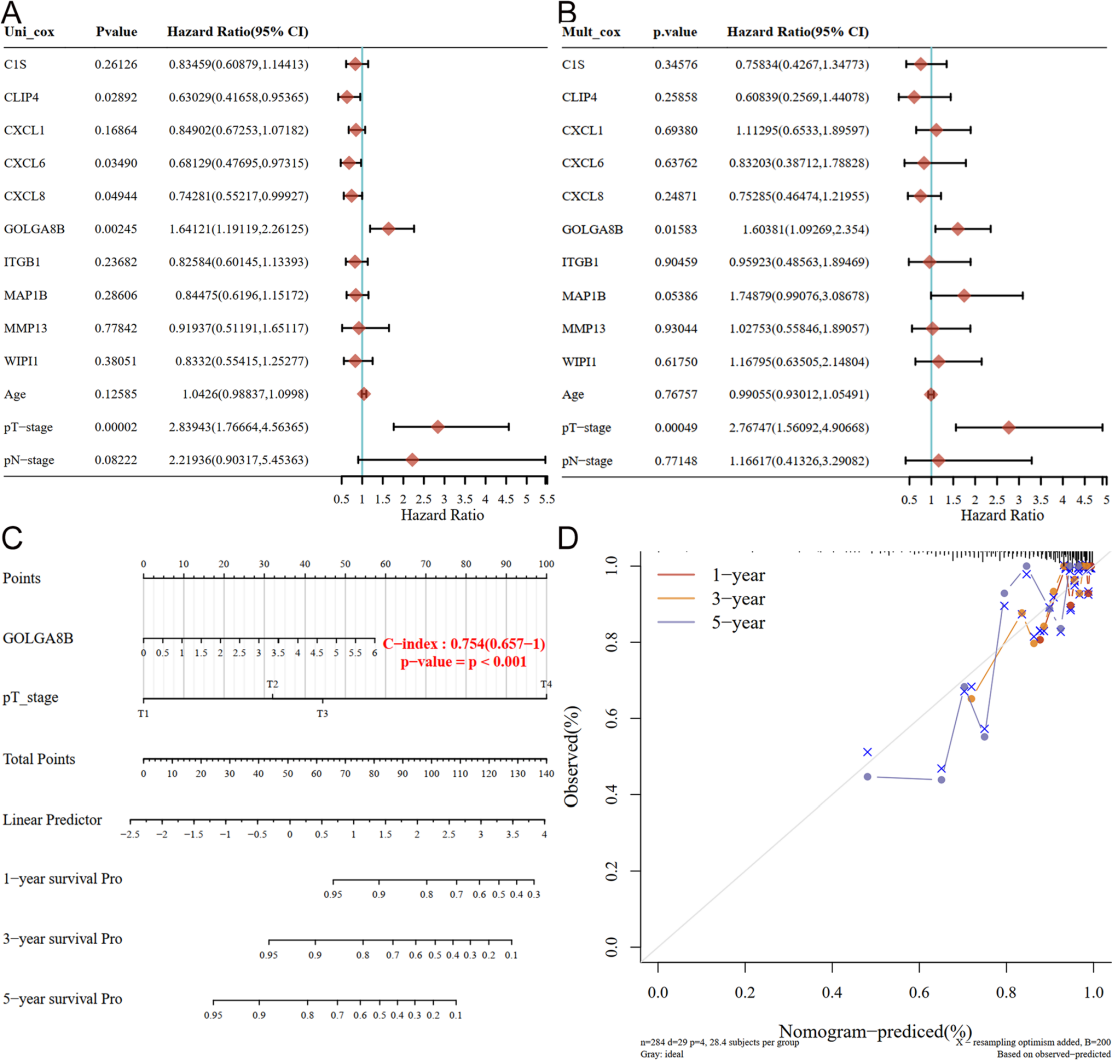
Cox regression reflects the risk of cabazitaxel resistance-related genes in influencing the DFS status of patients. **A** Single-factor and **B** muti-factor Cox regression analyses depict the role of cabazitaxel resistance-related genes in influencing the DFS status of patients. **C** Nomogram illustrates the function of cabazitaxel resistance-related genes in affecting the DFS status of patients. **D** Calibration curve of the nomogram

To ascertain the potential influence of these ten hub genes on PCa patient prognosis, survival clinical data from TCGA database were utilized. Single and multiple Cox regression models were developed to assess their impact on patient prognosis. In the single Cox regression model, *CXCL1*, *CXCL6*, *CXCL8*, and *GOLGA8B* were determined as genes influencing patient prognosis (*P* < 0.05; Fig. 5A). However, the multiple Cox regression model identified only *GOLGA8B* as a risk factor influencing patient prognosis (*P* < 0.05; Fig. 5B). Furthermore, a nomogram developed to predict the role of these cabazitaxel resistance-related genes in patient prognosis revealed that *GOLGA8B* was the only gene influencing prognosis (*P* < 0.001; Fig. 5C). Finally, a calibration curve was constructed to validate these results (Fig. 5D), reaffirming that *GOLGA8B* can impact the survival and prognosis of PCa patients.

### Association between GOLGA8B expression and immune cell infiltration in PCa

**Fig. 6:**
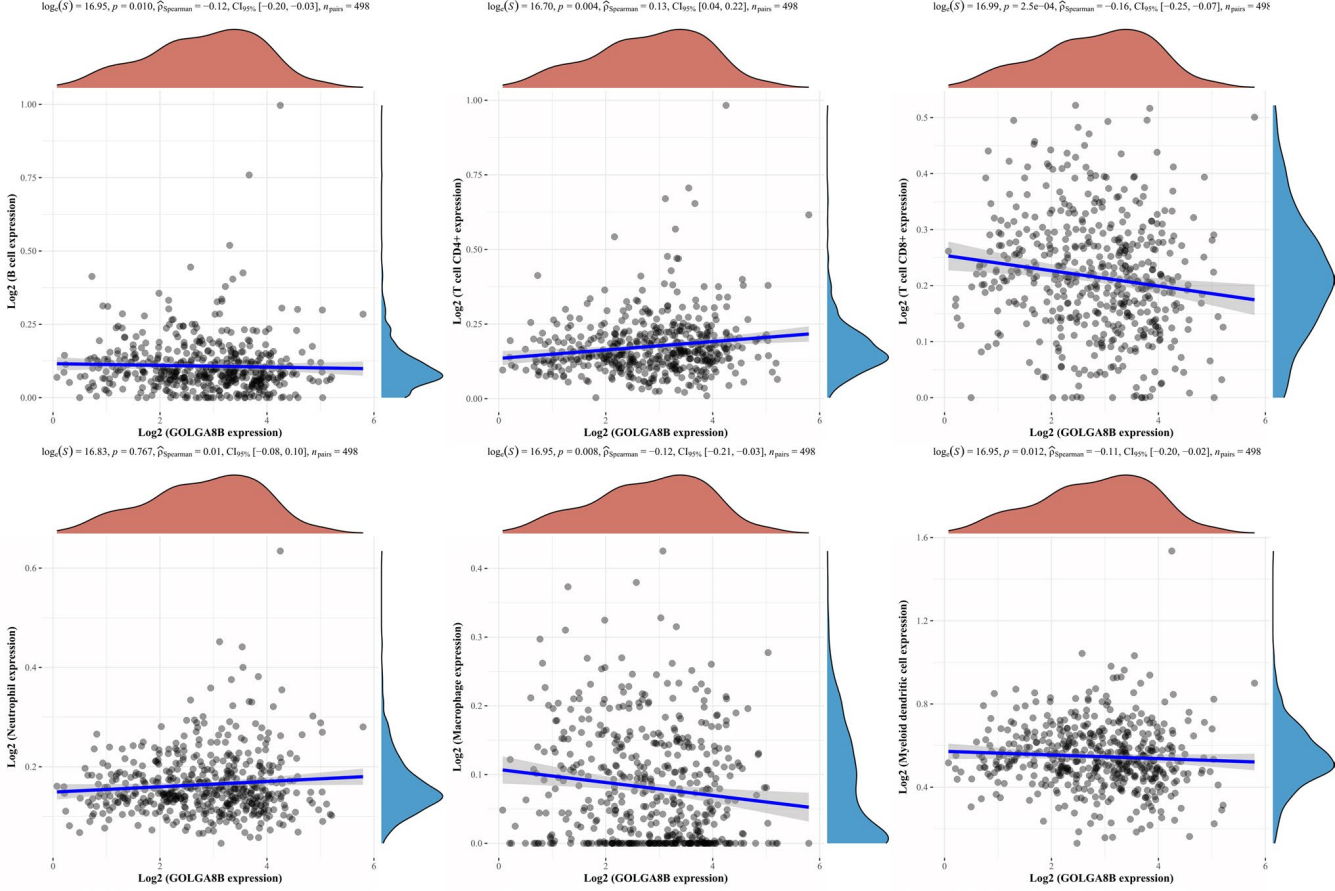
Correlation of *GOLGA8B* and immune cell infiltration depending on TCGA data

Infiltration of immune cells has been established to play a pivotal role in PCa progression [18]. Furthermore, cabazitaxel can affect breast cancer development by influencing immune cell infiltration [19]. Therefore, we hypothesized that cabazitaxel resistance-related genes are correlated with immune cell infiltration in PCa, contributing to its progression. Accordingly, we analyzed the correlation between cabazitaxel resistance-related genes and immune cell infiltration in PCa depending on the data from TCGA. The previous findings collectively emphasize the potential significance of *GOLGA8B* in PCa development and cabazitaxel resistance. Thus, we conducted an analysis to identify the correlation between some immune cells and *GOLGA8B* in PCa. Utilizing data from TCGA, we examined the association between *GOLGA8B* expression and various immune cell types within PCa. The analysis revealed a positive correlation between *GOLGA8B* expression and CD4^+^ T cells and neutrophils, whereas it demonstrated a negative correlation with B cells, CD8^+^ T cells, macrophages, and myeloid dendritic cells (Fig. 6). These results suggest that *GOLGA8B* might potentially contribute to PCa development and cabazitaxel resistance through its influence on immune cell infiltration.

### Expression of GOLGA8B in clinical PCa and CRPC samples

**Fig. 7:**
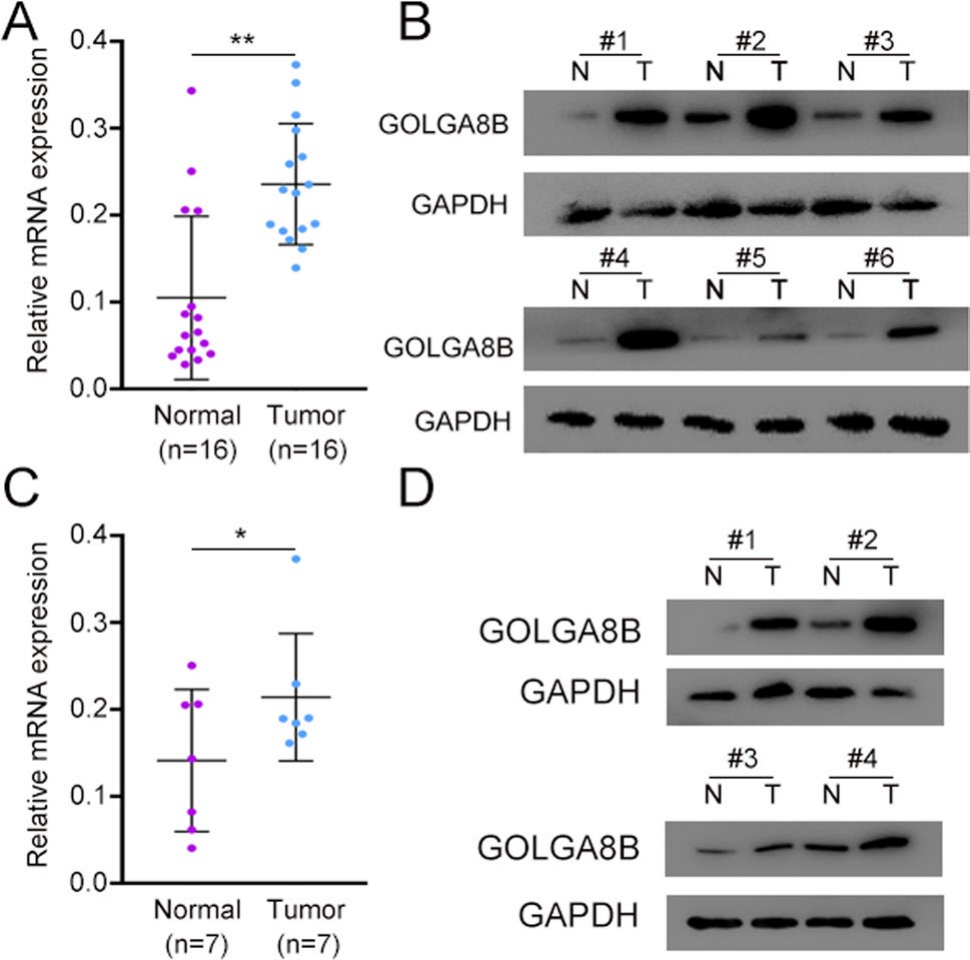
Expression of *GOLGA8B* in clinical PCa and CRPC samples. **A**, **B** mRNA and protein levels of GOLGA8B in normal tissues and PCa samples. **C**, **D** mRNA and protein levels of GOLGA8B in normal para-cancer tissues and CRPC samples. *P < 0.05, **P < 0.01. N: normal tissues, T: tumor tissues

We verified whether the expression of *GOLGA8B* changes upon the onset of PCa in clinical patients. For this purpose, we collected 16 paired tumor and pan-cancer normal tissues from clinical PCa patients, including seven CRPC patients. The clinical information is presented in Table support 1 (Table S1). In this study, we collected normal para-cancer and tumor tissues to assess GOLGA8B expression at both the mRNA and protein levels. The analysis revealed an increase in *GOLGA8B* expression levels *(P* < 0.01) in PCa tissues compared with that in normal tissues (Fig. 7A, B). Using clinical data of the patients involved, seven paired CRPC cases were selected for a subsequent evaluation of *GOLGA8B* expression. The results demonstrated an upregulation of GOLGA8B in CRPC tissues at both the mRNA and protein levels (*P* < 0.05; Fig. 7C, D). These findings underscore the pivotal role of *GOLGA8B* in the onset and progression of both PCa and CRPC.

### Impact of GOLGA8B on cabazitaxel sensitivity in CRPC cells

**Fig. 8:**
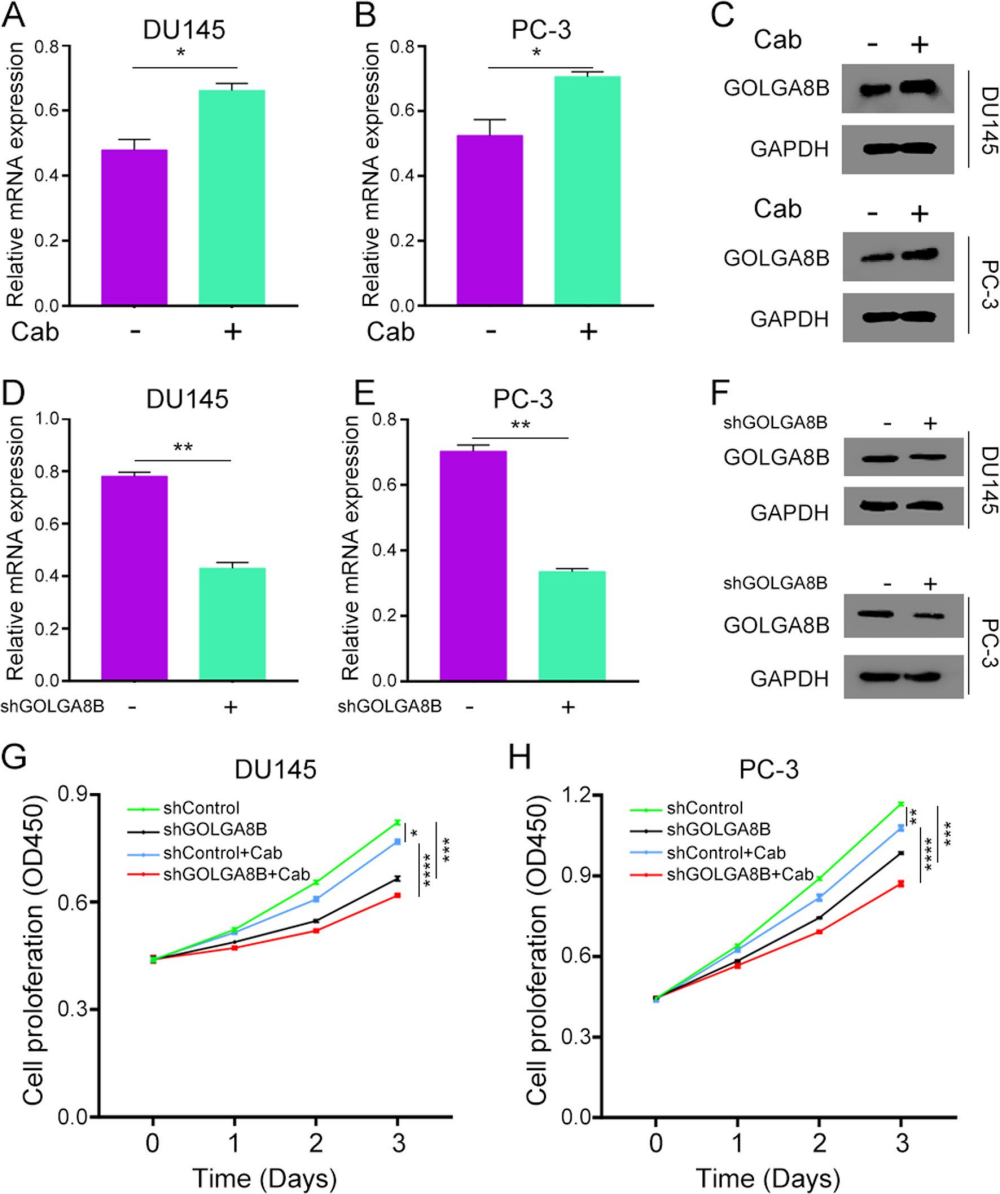
*GOLGA8B* is upregulated in cabazitaxel-resistant CRPC cells and affects the sensitivity of CRPC cells to cabazitaxel. **A**, **B** mRNA levels of *GOLGA8B* after a 24 h treatment of DU145 (**A**) and PC-3 (**B**) cells. **C** Protein levels of GOLGA8B after 24 h treatment of CRPC cells with cabazitaxel. **D**–**F** mRNA levels of *GOLGA8B* after transfection of DU145 (**D**) and PC-3 (**E**) cells with *GOLGA8B* knockdown (shGOLGA8B) lentivirus. **F** Protein levels of GOLGA8B after transfection of CRPC cells with shGOLGA8B lentivirus. **G**, **H** Cell proliferation ability of DU145 (**G**) and PC-3 (**H**) cells was assessed after transfection with shGOLGA8B lentivirus, or lack thereof, followed by treatment with cabazitaxel. *P < 0.05, **P < 0.01, ***P < 0.001, ****P < 0.0001. GADPH was used as an inner control in both qRT-PCR and western blotting. Cab: cabazitaxel

As *GOLGA8B* exhibited upregulation in cabazitaxel-resistant CRPC cells, it implied a potential role of this gene in CRPC resistance to cabazitaxel. Consequently, we evaluated how *GOLGA8B* affects the sensitivity of CRPC cells to cabazitaxel. After administering cabazitaxel to the DU145 and PC-3 cell cultures, an increase in the mRNA and protein expression levels of GOLGA8B was observed after a 24 h treatment period (*P* < 0.05; Fig. 8A–C). A *GOLGA8B* knockdown (shGOLGA8B) lentivirus was then constructed and administered to CRPC cells. The analysis demonstrated that shGOLGA8B lentivirus effectively reduced the expression of *GOLGA8B* in CRPC cells (*P* < 0.01; Fig. 8D–F). Next, we used a CCK-8 assay to detect the function of *GOLGA8B* in affecting the sensitivity of CRPC cells to cabazitaxel. We transfected the shGOLGA8B or shControl lentivirus into both DU145 and PC-3 cells and then treated them with cabazitaxel. We found that cabazitaxel decreased the proliferation of CRPC cells (*P* < 0.001; Fig. 8G, H). Additionally, we observed that CRPC cells transfected with shGOLGA8B lentivirus exhibited a decrease in cell proliferation ability (*P* < 0.05; Fig. 8G, H). We also found that cells with *GOLGA8B* knockdown were more sensitive to cabazitaxel (*P* < 0.0001; Fig. 8G, H). These findings underscore the involvement of *GOLGA8B* in modulating the sensitivity of CRPC cells to cabazitaxel, and cabazitaxel can effectively inhibit CRPC cell proliferation.

### Impact of GOLGA8B on docetaxel sensitivity in CRPC cells

**Fig. 9:**
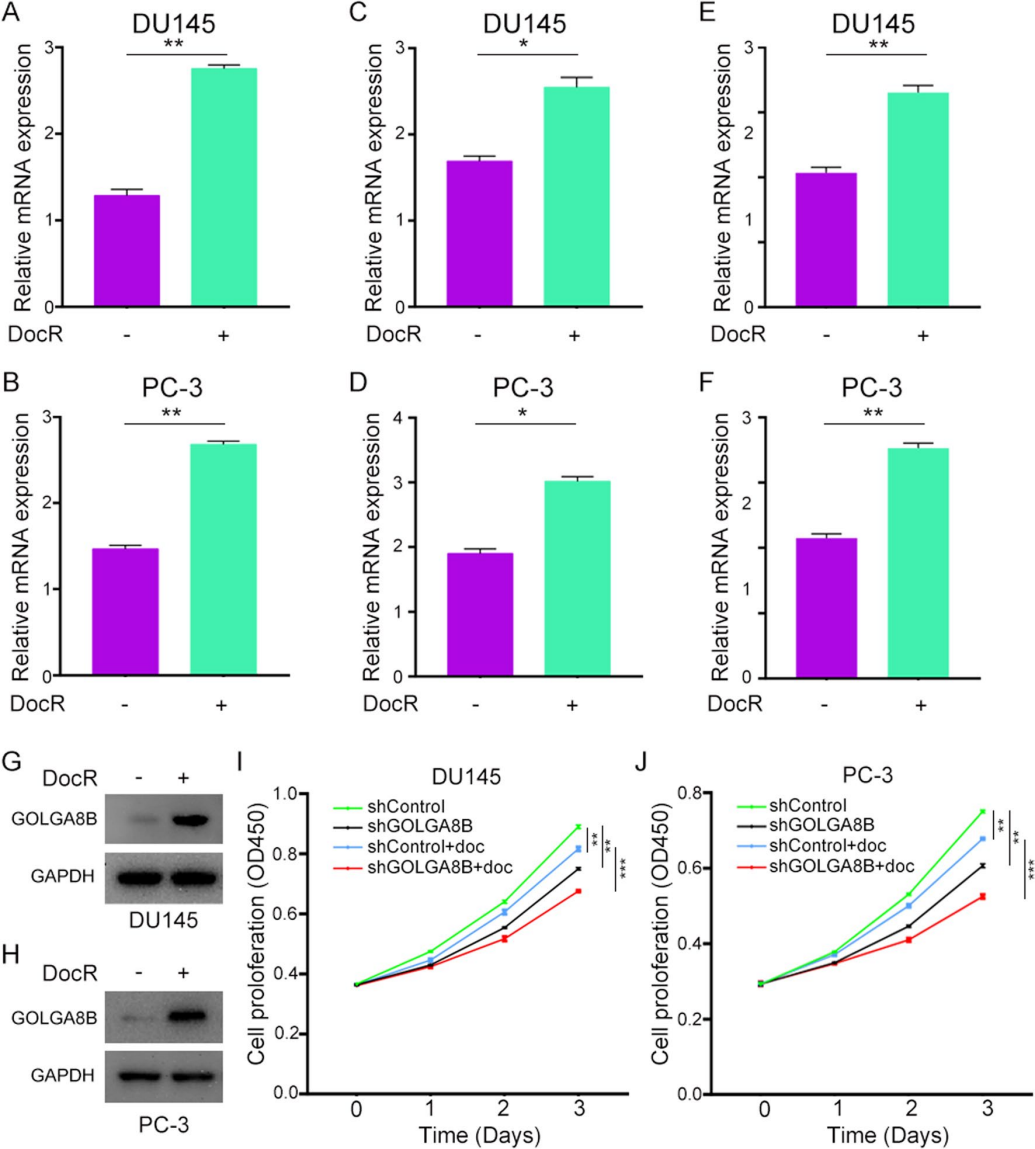
*GOLGA8B* is upregulated in docetaxel-resistant CRPC cells and affects the sensitivity of CRPC cells to docetaxel. **A**, **B** Expression of *GOLGA8B* in docetaxel-sensitive and -resistant DU145 (**A**) and PC-3 (**B**) cells from the GSE33455 dataset. **C**, **D** Expression of *GOLGA8B* in docetaxel-sensitive and -resistant DU145 **C** and PC-3 **D** cells from the GSE158494 dataset. **E**, **F** mRNA levels of *GOLGA8B* after 24 h treatment of DU145 (**E**) and PC-3 (**F**) cells with docetaxel. **G**, **H** Protein levels of *GOLGA8B* after 24 h treatment of DU145 (**G**) and PC-3 (**H**) cells with docetaxel. **I**, **J** Cell proliferation ability of DU145 (**I**) and PC-3 (**J**) cells treated by docetaxel after transfection with shGOLGA8B lentivirus, or lack thereof. *P < 0.05, **P < 0.01. GADPH was used as an inner control in both qRT-PCR and western blotting. Doc: docetaxel, DocR: docetaxel resistance

In the context of the primary use of cabazitaxel for treating docetaxel-resistant tumors, we investigated the impact of *GOLGA8B* on the sensitivity of CRPC cells to docetaxel. To achieve this, we utilized another dataset, GSE33455, containing microarray data of docetaxel-sensitive and -resistant CRPC cells. Analysis of this dataset revealed an upregulation of *GOLGA8B* in docetaxel-resistant CRPC cells compared with that in their docetaxel-sensitive counterparts (*P* < 0.01; Fig. 9A, B). Analysis of the GSE158494 dataset, containing data on docetaxel-resistant CRPC cells, revealed an increased expression level of *GOLGA8B* in these cells compared with that in the control (*P* < 0.05; Fig. 9C, D). Subsequently, we induced resistance to docetaxel in CRPC cells PC-3 and DU145 by treating them with docetaxel for 2 weeks. We observed that upon developing resistance to docetaxel, both PC-3 and DU145 cells exhibited an increase in GOLGA8B expression at both the mRNA and protein levels (*P* < 0.01; Fig. 9E–H). Furthermore, we transfected DU145 and PC-3 cells with shGOLGA8B or shControl lentivirus, treated them with docetaxel, and assessed their proliferation ability in the presence of docetaxel. The results indicated that the proliferation ability of CRPC cells decreased upon treatment with docetaxel (*P* < 0.01; Fig. 9I, J). Additionally, the data demonstrated that the knockdown of *GOLGA8B* rendered both DU145 and PC-3 cells highly sensitive to docetaxel (*P* < 0.001; Fig. 9I, J). Collectively, these findings highlight that, in addition to influencing sensitivity to cabazitaxel, *GOLGA8B* can impact the sensitivity of CRPC cells to docetaxel.

## Discussion

Due to the rapid increase in morbidity and mortality rates, PCa poses a significant threat to the health of older men worldwide [1]. Consequently, more effective treatments to enhance the survival of affected individuals are an immediate necessity. Various effective approaches have been employed for PCa treatment, with ADT being the primary option. Nevertheless, individuals undergoing ADT ultimately develop CRPC, and those entering this stage typically exhibit a median survival duration of less than 20 months [20].

Chemotherapy has demonstrated its effectiveness in treating CRPC. Drugs such as docetaxel and vinblastine have been employed in CRPC treatment for improving patient survival rates [13]. Docetaxel, as an efficacious therapeutic medication, can extend survival durations and enhance the efficacy of ADT in CRPC treatment [21, 22]. Nevertheless, individuals receiving docetaxel treatment may eventually develop resistance, necessitating the use of alternative second-line chemotherapy drugs.

Cabazitaxel, classified as a third-generation, semisynthetic taxane drug that binds to tubulin, is effective in treating tumors that have developed resistance to other taxanes [23]. A phase III open-label clinical trial in 2010 demonstrated the capacity of cabazitaxel to reduce the mortality risk in patients with CRPC following the development of docetaxel resistance [11]. Additionally, another study reported that cabazitaxel can delay tumor progression and enhance the survival rates of CRPC patients [12]. These findings highlight cabazitaxel as an effective treatment option for CRPC, despite potential adverse effects in carcinoma treatment [24]. Nevertheless, resistance to cabazitaxel can also emerge over time in patients previously responsive to the drug. Thus, investigating the potential mechanisms underlying CRPC resistance to cabazitaxel holds promise for improving CRPC management and treatment.

Genetic alterations have been established as pivotal contributors to PCa resistance to chemotherapy drugs. Several studies have indicated that genetic alterations represent a crucial factor in PCa resistance to chemotherapeutic agents, such as docetaxel and vinblastine [13–15]. Furthermore, additional research has revealed genetic alterations as an underlying cause of PCa resistance to cabazitaxel. The upregulation of *TUBB3* in PCa cells reportedly leads to resistance to cabazitaxel [16]. Furthermore, the amplification of *ABCB1* can cause CRPC cells to become resistant to cabazitaxel [17]. These results indicate that genetic changes are crucial for PCa resistance to cabazitaxel. However, until now, systematic studies on hub genes important for CRPC resistance to cabazitaxel are lacking.

In 2020, a study utilized next-generation RNA sequencing to comprehensively compare genes with differential expression between cabazitaxel-sensitive and -resistant CRPC cells [25]. Through the analysis of this study, hub genes were identified as potentially significant contributors to cabazitaxel resistance in CRPC. Furthermore, an examination of their roles in influencing PCa progression was performed. Within the analysis, the upregulation of ten hub genes in cabazitaxel-resistant CRPC cells was observed. Additionally, these genes, associated with cabazitaxel resistance, demonstrated an impact on the onset and progression of both PCa and CRPC. A pivotal gene, *GOLGA8B*, was identified as capable of influencing the survival and prognosis of patients with PCa. Lastly, upregulation of *GOLGA8B* was observed in patients with PCa or CRPC, and it was found to affect the sensitivity of CRPC cells to both cabazitaxel and docetaxel.

*GOLGA8B* is associated with various diseases, and alterations in *GOLGA8B* expression may contribute to the development of conditions such as dementia and coronary atherosclerosis [26, 27]. Additionally, *GOLGA8B* expression has been linked to the occurrence of multiple types of tumors. For instance, one study revealed that *GOLGA8B* can promote lung squamous cell carcinoma by suppressing the expression of *STAT3* [28]. Furthermore, certain long non-coding RNAs play a role in the development of clear cell renal cell carcinoma by regulating *GOLGA8B* expression levels [29]*.* Another study, utilizing bioinformatics approaches, highlighted the potential significance of *GOLGA8B* in PCa [30]. In our study, we observed that *GOLGA8B* not only influences PCa progression and prognosis but also serves as a key gene in conferring resistance against both cabazitaxel and docetaxel in CRPC.

Nevertheless, our study has certain limitations. First, the data on cabazitaxel-resistant CRPC were obtained solely from cell lines within the GSE158494 dataset rather than from clinical samples. The information derived from cell lines alone may substantially differ from clinical data, and relying on a single dataset may introduce bias. However, collecting clinical cabazitaxel-resistant CRPC samples is extremely challenging, limiting our analysis to cell lines. Consequently, a possibility of incompleteness exists in identifying cabazitaxel resistance-related genes, and bias could be present. Second, while assessing *GOLGA8B* expression in clinical PCa and CRPC samples, the sample size was small. Therefore, the findings may not be fully representative, and the results require validation with a larger sample size. Third, although *GOLGA8B* was ascertained to play a pivotal role in CRPC resistance to cabazitaxel, the precise underlying mechanism remains unclear. Consequently, future research should prioritize elucidating the mechanism via which *GOLGA8B* promotes CRPC resistance to cabazitaxel.

## Conclusion

In this study, we identified ten hub genes that may play important roles in cabazitaxel resistance in CRPC. Furthermore, *GOLGA8B* was demonstrated to play a pivotal role in the onset and progression of CRPC and serve as a key regulator of cabazitaxel resistance in CRPC.

### Supplementary Information

Additional file1 (DOCX 14 KB)

Additional file 2 (TIF 3244 KB)

## Data Availability

The datasets supporting the conclusions of this article are available in the public databases such as GEO, TCGA and CPGEA. Other data can get from the correspondence author.
