# Supplementary material for: Exploration of the influence of GOLGA8B on prostate cancer progression and the resistance of castration-resistant prostate cancer to cabazitaxel
Source: Discov Oncol. 2024 May 10;15:152. doi: 10.1007/s12672-024-00973-7 (PMC11087400; doi:10.1007/s12672-024-00973-7)
Supplement: Supplementary file 1 — Additional file1 (DOCX 14 KB) [file 12672_2024_973_MOESM1_ESM.docx]

Table S1: The clinical information of patients included in the study

| Name | Age | Age in diagnosis | TNM | Gleason Score | CRPC |
| --- | --- | --- | --- | --- | --- |
| Wang✳✳ | 74 | 70 | T2N1M0 | 3+4 | Yes |
| Li ✳✳ | 78 | 73 | T2N0M0 | 3+4 | Yes |
| Chen ✳ | 86 | 82 | T3N0M0 | 4+4 | Yes |
| Ling ✳ | 82 | 80 | T2N0M0 | 3+4 | No |
| Liu ✳✳ | 83 | 81 | T2N0M0 | 3+4 | No |
| Wu ✳✳ | 75 | 73 | T3N1M0 | 5+4 | No |
| Jin ✳✳ | 77 | 74 | T4N0M0 | 4+4 | Yes |
| Gu ✳✳ | 69 | 65 | T2N0M0 | 3+4 | Yes |
| Shen ✳ | 72 | 70 | T3N0M0 | 3+4 | No |
| Shi ✳✳ | 87 | 85 | T3N1M0 | 4+4 | No |
| Meng ✳ | 66 | 62 | T4N1M1 | 5+4 | Yes |
| Zhao ✳ | 86 | 82 | T3N0M0 | 4+3 | Yes |
| Pan ✳✳ | 80 | 78 | T2N0M0 | 4+3 | No |
| Zhao ✳ | 73 | 71 | T3N0M0 | 4+4 | No |
| Liu ✳ | 81 | 79 | T2N0M0 | 3+4 | No |
| Yang ✳ | 74 | 73 | T3N0M0 | 4+4 | No |
