# Supplementary figures and images for: Exploration of the influence of GOLGA8B on prostate cancer progression and the resistance of castration-resistant prostate cancer to cabazitaxel
Source: Discov Oncol. 2024 May 10;15:152. doi: 10.1007/s12672-024-00973-7 (PMC11087400; doi:10.1007/s12672-024-00973-7)

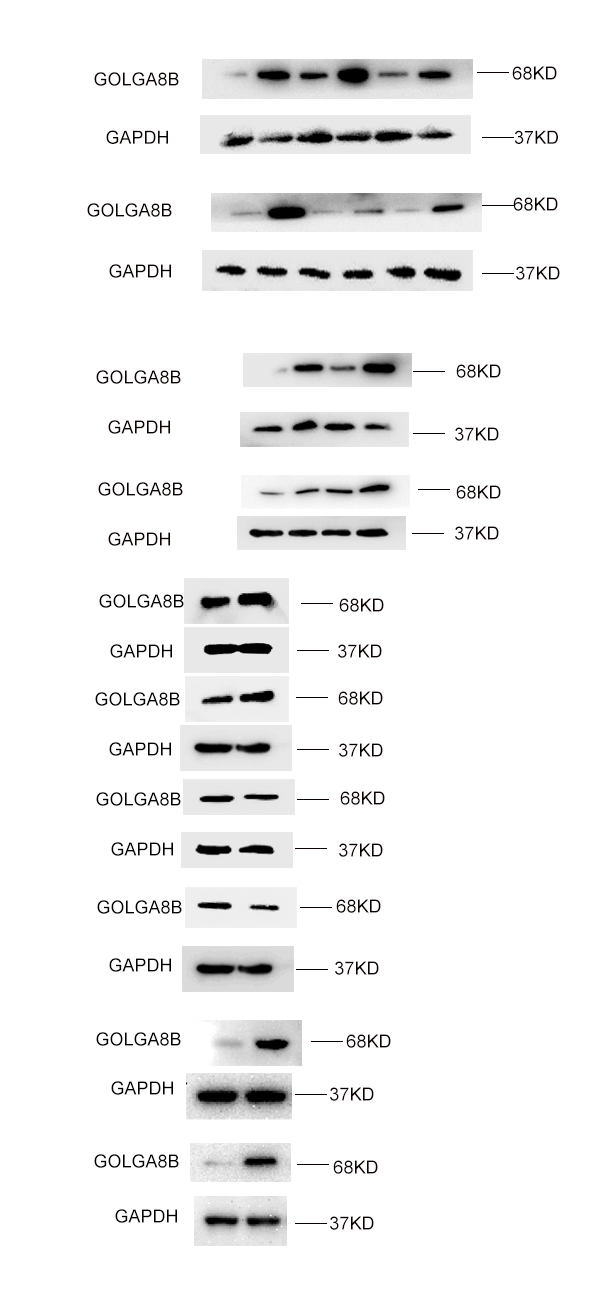

Supplement: Supplementary file 2 — Additional file 2 (TIF 3244 KB) [file 12672_2024_973_MOESM2_ESM.tif]
